# Benefits of a mosaic approach for assessing cortical atrophy in individual multiple sclerosis patients

**DOI:** 10.1002/brb3.3327

**Published:** 2023-11-13

**Authors:** Marlene Tahedl, Tun Wiltgen, Cui Ci Voon, Achim Berthele, Jan S. Kirschke, Bernhard Hemmer, Mark Mühlau, Claus Zimmer, Benedikt Wiestler

**Affiliations:** ^1^ Department of Neuroradiology, School of Medicine Technical University of Munich Munich Germany; ^2^ Department of Neurology, School of Medicine Technical University of Munich Munich Germany

**Keywords:** biomarker, gray matter atrophy, individual MRI, MAP, mosaic‐based approach, multiple comparisons, relapsing‐remitting multiple sclerosis, smoothing

## Abstract

**Objective:**

Cortical gray matter (GM) atrophy plays a central role in multiple sclerosis (MS) pathology. However, it is not commonly assessed in clinical routine partly because a number of methodological problems hamper the development of a robust biomarker to quantify GM atrophy. In previous work, we have demonstrated the clinical utility of the “mosaic approach” (MAP) to assess individual GM atrophy in the motor neuron disease spectrum and frontotemporal dementia. In this study, we investigated the clinical utility of MAP in MS, comparing this novel biomarker to existing methods for computing GM atrophy in single patients. We contrasted the strategies based on correlations with established biomarkers reflecting MS disease burden.

**Methods:**

We analyzed T1‐weighted MPRAGE magnetic resonance imaging data from 465 relapsing‐remitting MS patients and 89 healthy controls. We inspected how variations of existing strategies to estimate individual GM atrophy (“standard approaches”) as well as variations of MAP (i.e., different parcellation schemes) impact downstream analysis results, both on a group and an individual level. We interpreted individual cortical disease burden as single metric reflecting the fraction of significantly atrophic data points with respect to the control group. In addition, we evaluated the correlations to lesion volume (LV) and Expanded Disability Status Scale (EDSS).

**Results:**

We found that the MAP method yielded highest correlations with both LV and EDSS as compared to all other strategies. Although the parcellation resolution played a minor role in terms of absolute correlations with clinical variables, higher resolutions provided more clearly defined statistical brain maps which may facilitate clinical interpretability.

**Conclusion:**

This study provides evidence that MAP yields high potential for a clinically relevant biomarker in MS, outperforming existing methods to compute cortical disease burden in single patients. Of note, MAP outputs brain maps illustrating individual cortical disease burden which can be directly interpreted in daily clinical routine.

## Graphical abstract

Different strategies for assessing individual cortical disease burden in multiple sclerosis (MS) were compared. A mosaic approach (MAP), based on referencing parcelled individual CT to control data, was previously shown to be clinically relevant in motor neuron diseases (MND) and frontotemporal dementia (FTD) and was now evaluated for MS. MAP yielded highest correlations with clinical disability assessment scores in MS and thus yields high potential for a biomarker capturing clinically relevant cortical disease burden in MS, thereby highlighting the generalizability of MAP beyond MND and FTD.

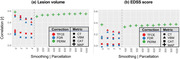

## 1 INTRODUCTION

### 1.1 Relevance of appreciating cortical disease burden in multiple sclerosis patients and the “MAP” approach

Multiple sclerosis (MS) is a debilitating disorder of the central nervous system (CNS) which remains incurable until today (Lassmann, 2018). The most prominent pathological substrate of MS pathology are lesions to the white matter (WM) of the CNS, which present as hyperintensities on T2‐weighted FLAIR magnetic resonance images (MRI). Although WM lesion load does explain variance in terms of clinical disease burden—in particular cognitive functioning (Engl et al., 2020; Mollison et al., 2017) and its progression are negatively correlated with cognitive decline (Todea et al., 2020)—it has become clear that it does not capture the entirety of the clinical state (Barkhof, 2002; Popescu et al., 2013). Advances in neuroimaging methods allowed to identify another and at least partly independent anatomical substrate, namely, the shrinking and/or decay of CNS cells, or atrophy (Eshaghi et al., 2018; Filippi & Rocca, 2010; Vrenken & Geurts, 2007). Atrophy has been shown to be even more closely related to disability progression than WM lesion load (Bakshi et al., 2001; de Stefano et al., 2003; Sailer et al., 2003).

In our previous work, we have suggested to estimate individual gray matter (GM) cortical atrophy based on rating single subject's parceled cortical thickness (CT) data and evaluating the single “mosaics” with respect to healthy controls (HC) (Tahedl, 2020). In a series of studies, we have evaluated and demonstrated the utility of mosaic approach (MAP) for assessing clinically relevant cortical disease burden in motor neuron diseases (MND) and frontotemporal dementia (FTD) (see, Section 1.4). In this study, we aim to probe the clinical utility of MAP in MS, comparing this novel biomarker to existing methods for computing GM atrophy in single patients.

### 1.2 Assessing individual deep gray matter and white matter disease burden

Atrophy of both deep GM nuclei as well as cortical atrophy reflect aspects of MS disability but might cover distinct features (Eijlers et al., 2019). However, both forms of atrophy do inform the clinical state, such that taking into account both markers—in addition to the well‐established biomarker WM lesion load, as discussed above—should provide a more complete assessment of the individual patient's clinical state and might allow for more accurate prognoses. For effective clinical translation, such a biomarker needs to be readily acquirable and interpretable in a personalized manner.

For WM lesions and deep GM atrophy, the assessment of an individual estimate is relatively straightforward using advanced neuroimaging methods: In order to quantify WM lesion load (and thus allowing long‐term monitoring), (half‐)automated software‐supported methods have been developed that can be applied to single subjects’ T1w/T2‐FLAIR data sets which output volume estimates of the individual lesion load with high accuracy (Mortazavi et al., 2012; Schmidt et al., 2012). Similarly, automated segmentation methods exist that provide reliable volume estimates for distinct deep GM nuclei (Fischl et al., 2002, 2004; Mendelsohn et al., 2023).

### 1.3 Assessing individual cortical disease burden

Unlike WM lesion load and subcortical volumetry, GM atrophy is much harder both to quantify and to interpret on a single‐patient level (Amiri et al., 2018). In terms of quantification, one problem is that cell damage or death induces a complex process of tissue restructuring which can hardly be reflected by a singular metric (Tsouki & Williams, 2021). Therefore, the neuroimaging community has suggested a wide number of metrics to quantify cortical GM changes, including the absolute CT (e.g., Fischl & Dale, 2000), its volume (e.g., Gaser et al., 2022) as well as “hybrids” such as GM concentration (most famously via the voxel‐based morphometry [VBM], VBM, approach, e.g., Ashburner & Friston, 2000). All of these metrics have been demonstrated to mirror disease burden in MS on the group level; nevertheless, attempts to engineer metrics from them that can be applied to single subjects remain a challenge (Amiri et al., 2018).

### 1.4 Challenges of existing methods for single‐patient assessment of GM atrophy

The problem of constructing clinically relevant biomarkers for cortical atrophy is aggravated by the fact that neuroimaging pipelines regularly offer to adjust a wide range of parameters in both pre‐ and postprocessing of data which can impact downstream analysis results (Cash et al., 2015; Gunter et al., 2003; Popescu et al., 2016) and impede widespread adoption and generalization. The rationale behind many of those parameters is to increase statistical power of the analysis—it needs to be emphasized that in neuroimaging, the cortex is typically represented as hundreds of thousands of distinct data points. This requires rigorous control of the multiple comparisons problem, which can easily hamper statistical power if using common correction methods such as suggested by Bonferroni (Simes, 1986). One way to address this problem is to smooth the data before running statistics to reduce the variability between neighboring data points (Jo et al., 2007)—however, notice that there is no clear consensus on the extent of “optimal” smoothing. Another strategy is to choose from less rigorous family‐wise error rate (FWER) correction methods, tailored to neuroimaging data, such as threshold‐free cluster enhancement (TFCE, Smith & Nichols, 2009) or employ alternative approaches, for example, the false discovery rate (FDR, Benjamini & Hochberg, 1995; Genovese et al., 2002).

### 1.5 Employing a mosaic approach (MAP) to assess clinically relevant cortical disease burden

The above list of choices to identify cortical GM changes is far from being complete but indicates how one might easily get overwhelmed by the abundant number of possible combinations throughout data analysis. Moreover, there is no clear directive on how adjustment of the technical details and their combinations impacts downstream results, which in turn hampers interpretability. This might be one reason why the development of a readily accessible biomarker for interpreting single‐patient GM atrophy is still lacking in MS.

To overcome this gap, we have developed the “MAP” (Tahedl, 2020), which assesses individual cortical disease burden for distinct brain regions (“mosaics”). The primary outcome variable of MAP is a single number reflecting the fraction of potentially atrophic cortex (referred to as “thin‐patch fraction”). In a series of studies, we have evaluated and demonstrated the utility of MAP for assessing clinically relevant cortical disease burden in MND and FTD: More specifically, we could demonstrate that MAP reflects both the cross‐sectional as well as the longitudinal progression of clinical disability in amyotrophic lateral sclerosis (ALS), as measured by the ALS Functional Rating Scale, revised (Tahedl et al., 2021). Moreover, we could show that MAP accurately reflects the histopathologic involvement of cortical disease burden: By contrasting (1) a purely upper motor neuron—that is, purely cortical—condition, namely, primary progressive lateral sclerosis (PLS), (2) a mixed upper‐/lower motor neuron condition (ALS), and (3) a primary lower motor neuron condition (poliomyelitis survivors), we could show that this degree of cortical involvement is captured by MAP—both cross‐sectionally and longitudinally (Tahedl et al., 2022). Furthermore, we have investigated the clinical utility of MAP outside of MND and applied it to the FTD spectrum, where we could also demonstrate its clinical utility for single patients (McKenna et al., 2022).

### 1.6 MAP—a novel biomarker for assessing individual atrophy also in MS?

**FIGURE 1:**
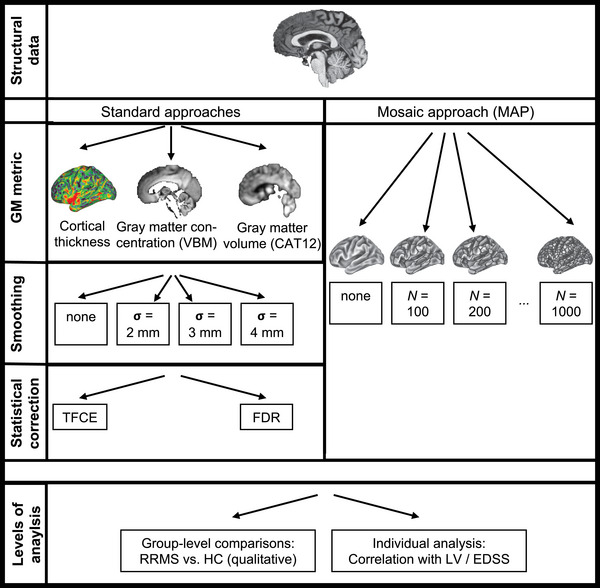
**Study objective**. The current work contrasted different strategies for assessing gray matter (GM) variations in relapsing‐remitting multiple sclerosis (RRMS) patients. In specific, we compared different metrics for calculating GM differences (cortical thickness [CT], GM concentration via VBM, GM volume via CAT12), smoothing (no smoothing, *σ* = 2/3/4 mm), and correction methods for multiple comparisons (threshold‐free cluster enhancement [TFCE]‐ vs. false discovery rate [FDR]‐correction). In addition, we considered the mosaic approach (MAP), a method to assess CT differences based on parcellation the cortex into smaller subregions and, for individual patients, calculating whether or not that parcel was significantly thin as compared to a healthy control (HC) control group. We evaluated both group‐level comparisons (by inspecting statistical output maps between the combinations) as well as individual‐level comparisons (by investigating which combination correlated best with lesion volume [LV] on the one hand and expanded disability status score [EDSS] on the other hand). CAT12, computational anatomy toolbox, version 12 (used for calculating GM volume); VBM, voxel‐based morphometry (used for estimating GM concentration).

Our previous investigations into MND and FTD motivate us to interrogate the utility of MAP also in MS, given the need for an individual cortical biomarker in that condition as outlined above. In this study, we provide a thorough investigation of MAP utility in MS. Importantly, we focus our study on comparisons with existing strategies to compute individual cortical disease burden (which we refer to as “standard approaches”) and technical variations of those (including smoothing and statistical correction methods, Figure 1). We also consider variations of MAP by contrasting different parcellation schemes and varying the resolution of the respective partitioning. We appreciate both effects on individual‐ and group‐level statistical comparisons between the methods. Moreover, we validate the clinical utility of the approaches based on their associations with correlations to third metrics reflecting clinical disease burden in MS, namely, WM lesion load and Expanded Disability Status Scale (EDSS) scores, which both reflect at least partly independent and complementary information on the MS disease course/progression (Sormani et al., 2014). With this careful inspection, we aim to explore the benefits of MAP for assessing clinically relevant cortical disease burden for single MS patients, thereby extending MAP generalizability beyond MND and FTD. Indeed, MAP outputs brain maps that illustrate single‐subject cortical involvement, which can be used for personalized assessment and monitoring in MS clinical routine. We end our investigation with a demonstration of such single‐subject visualization.

## 2 METHODS

### 2.1 Subjects

In total, 465 relapsing‐remitting MS (RRMS) patients and 89 HC were included in this study (Table 1). Patients were diagnosed according to the 2017 revisions of the MacDonald criteria (Thompson et al., 2018). 3D T1w MPRAGE images were acquired at the Klinikum rechts der Isar of the Technical University of Munich on one of two 3.0 T whole body Philips MRI scanners (1) *Achieva dStream*, (2) *Ingenia* with identical scanning parameters: 267 sagittal slices, field of view = 240 × 252 mm^2^, spatial resolution = 1.00 mm^3^, repetition time = 9 ms, echo time = 4 ms, flip angle = 8°, and acquisition time = 2 min 25 s. Moreover, demographic data, including age and gender were recorded. For patients, additionally disease duration, EDSS scores and the dominant hand were assessed at a time point close to the MR session (±1 week) by a certified neurologist. All subjects had given informed consent to the use of their data for research purposes. The study procedures followed safety guidelines for MRI research at the Technical University of Munich, which are in line with the Declaration of Helsinki and were approved by the local ethics committee. Demographic variables including age means and gender distributions between RRMS and HC were performed using Welch's two‐sample *t*‐tests and Chi‐squared test with Yate's continuity correction.

**TABLE 1:** Demographic details of the study populations.

	RRMS patients	HC	Group differences (W^+^/C^2++^)
Total number of subjects	465	89	n.a.
Age [y, mean ± SD]	40.14 ± 9.94	37.36 ± 15.06	W: *t*(103.16) = 1.67, *p* = .10
Sex, F/M	305/160	59/30	C^2^ *: χ* ^2^(1, *N* = 554) = 3.27e − 5, *p* = .995
Dominant hand, R/L	408/57	n.a.	*n.a*.
Average whole brain cortical thickness [mm, mean ± SD]	2.35 ± 0.09	2.41 ± 0.11	W: *t*(113.31) = −4.60, *p* < .001^*^
Lesion volume [mL, mean ± SD]	5.39 ± 8.41	n.a.	n.a.
EDSS score [median (IQR)]	1.50 (2.00)	n.a.	n.a.
Disease duration [y, mean ± SD]	8.65 ± 5.16	n.a.	n.a.

*Note*: ^+^ = Welch two‐sample *t*‐tests were performed to test differences of age and years of education between all patients versus HC, ^++^ = Chi‐square tests were performed to test differences of sex and handedness frequencies between all patients versus HC.

Abbreviations: C^2^, Chi‐square test; EDSS, expanded disability status score; F, female; HC, healthy control; L, left‐handed; M, male; ml, milliliter; MRI, magnetic resonance imaging; MS, multiple sclerosis; N, sample size; n.a., not applicable; R, right‐handed; RRMS, relapsing‐remitting MS; SD, standard deviation; W, Welch two‐sample *t*‐test; y, years.

^*^Significant at an alpha‐level of *p* ≤ .05.

### 2.2 Neuroimaging data analysis

#### 2.2.1 Preprocessing

The idea of this study was to evaluate and compare three different commonly used strategies for assessing GM variations, as well as to document impacts of smoothing and methods to correct for the multiple comparisons problem (Figure 1). As such, we preprocessed structural data for all subjects using three different analysis pipelines to calculate (a) CT, (b) VBM, which reflect aspects related to GM concentration, and (c) GM volume (using the computational anatomy toolbox [CAT12]). To calculate CT, we started with a full image segmentation and surface reconstruction using FreeSurfer's *recon‐all* pipeline (Dale et al., 1999; Fischl, 2012; Fischl et al., 1999). These data were then converted to the CIFTI format to improve visualization and downstream data handling (Dickie et al., 2019). The main output from this pipeline for our study was a file which estimates CT at each of 32k vertices per hemisphere for each subject. VBM was calculated within FMRIB's Software Library (FSL) (Jenkinson et al., 2012), after running its structural processing algorithm *fsl_anat*. We calculated voxelwise VBM maps for each patient within FSL (using the software's standard recommendations, Good et al., 2002). Finally, GM volume was assessed using the CAT12 toolbox from the SPM12 software package, also following the manual's standard guidelines (Schmidt et al., 2012). Both VBM and CAT12 data were then warped to the MNI152 2 mm standard space to enhance group‐level comparisons. Finally, WM lesion segmentation was performed using the lesion growth algorithm of LST toolbox, which outputs an estimate of a given subject's total lesion volume (LV), among others.

### 2.3 Smoothing

One goal of this study was to document effects of different smoothing factors on the preprocessed data on statistical comparisons. In brief, the rationale behind smoothing is to account for inaccuracies in spatial registration as well as increase the signal‐to‐noise ratio (SNR, e.g., Hopfinger et al., 2000, who published a comprehensive analysis of smoothing effects on fMRI data). In total, we contrasted four different smoothing factors, namely, (a) no smoothing (*σ* = 0 mm), (b) *σ* = 2 mm, (c) *σ* = 3 mm, and (d) *σ* = 4 mm. Notice that commonly, smoothing factors are provided as full‐width at half maximum (FWHM) Gaussian kernels, which can be directly calculated from sigma by multiplying with a factor of approximately 2.3548, such that, for example, *σ* = 2 mm translates into a Gaussian kernel FWHM = 4.7096 mm. We applied those smoothing factors to the outputs of all three structural preprocessing pipelines, whereas we used tools from Workbench (Glasser et al., 2013; Marcus et al., 2011) for the surface‐based CT data and tools from FSL (Jenkinson et al., 2012) for smoothing volumetric‐based data, that is, VBM and CAT.

### 2.4 Cortical parcellation

As specified above, one rationale behind smoothing is to boost SNR and therefore statistical power. Alternatively, one can increase power by reducing the number of statistical comparisons, for example, by averaging across neighboring data points (Tahedl, 2020). This “parcellation method” has been suggested previously for CT data and has been demonstrated to yield clinically relevant information for various neurological conditions such as ALS (Tahedl et al., 2021) and FTD (McKenna et al., 2022). However, these studies did not interrogate effects of different CT parcellation schemes but have used a cortical parcellation of *N* = 1000 “patches” (in total for both hemisphere), using predefined and roughly equally sized atlas regions (Schaefer et al., 2018). Here, we considered 10 different parcellation schemes (from *N* = 100 to 1000 patches in steps of *N* = 100), as well as no parcellation (i.e., each vertex is its own “patch,” such that *N* = 59,234 patches). The parcellation schemes were taken from work by Schaefer et al. (2018), analogous to the original studies suggesting the parcellation method.

### 2.5 Group‐level statistics

#### 2.5.1 Study design: “Standard approaches”

**FIGURE 2:**
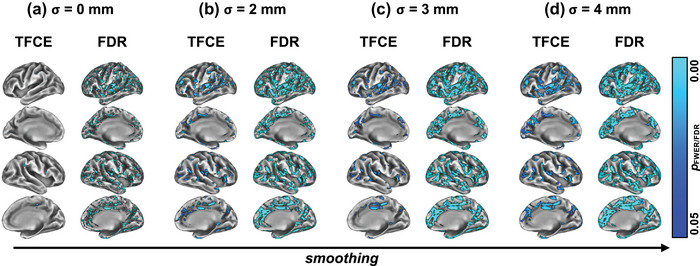
**Group‐level comparison of cortical thickness (CT)**. Contrasting CT between relapsing‐remitting multiple sclerosis (RRMS) and healthy control (HC) for different neuroimaging parameters, we found that family‐wise error (FWE)‐correction yields “cleaner” output maps as compared to false discovery rate (FDR)‐corrected statistical maps (compare left vs. right columns on A–D). In addition, no smoothing (a) almost suppressed any significant results for FWE‐correction, smoothing kernels of *σ* = 2 mm (b), 3 mm (c), and 4 mm (d) produced clearly defined maps of gray matter (GM) variations, centered around the left, and right insular/temporal cortices. TFCE, threshold‐free cluster enhancement.

We were interested in effects of different “standard approaches” to assess GM variations both on group‐level statistical comparisons as well as individual‐versus‐group statistical comparisons. All statistics (but for the CT parcellation, see below) were run within FSL using it is *randomize* tool (notice that CT data were converted temporarily to NIFTI data using tools from Workbench to ensure comparability with FSL algorithms). For all three standard approaches of assessing GM alterations, we limited our contrasts to a GM cortical mask (see Figure 2A). We used general linear models (GLM), corrected for age and gender, for both group‐level and individual‐versus‐group statistics. For the group‐level statistics, we set up the design matrix as a two‐group difference comparison, whereas for the individual‐versus‐group comparisons we set up the design matrix as “Singleton‐versus‐Group” or “Prediction‐Interval‐Test” (following FSL's recommendations/terminology on setting up GLMs), which is basically the same test as specified for the group contrast when one group has exactly one subject in it, that is, in our case the respective RRMS patient (which was tested against *N* = 89 HC and run for each patient separately). Both comparisons were adjusted for the covariates age and gender. As we were merely interested in GM variations indicating atrophy, we ran one‐sided testing and considered only the contrast *MS < HC*.

#### 2.5.2 Correction for multiple comparisons: “Standard approaches”

We contrasted two common ways to account for multiple testing for standard approaches, namely, (a) TFCE and (b) FDR. Although TFCE can be directly applied with FSL's *randomize* (notice that we used the algorithm's default settings, that is, 5000 permutations, among others), FDR‐correction can be run on the p‐maps uncorrected for multiple comparisons, which can be also output from *randomize*. Also, for latter correction, we used FSL's default settings (i.e., voxelwise‐thresholding at *p* ≤ .05, 5000 permutations).

#### 2.5.3 Study design: mosaic approach (MAP)

The study design for the MAP, that is, parcellating CT data and evaluating it with respect to HC, deviated from the standard approaches given the rationale behind this strategy (which was to assess whether a single subject's patch is “significantly thin” as compared to a reference group). As suggested in the original MAP paper (Tahedl, 2020), we started by averaging all vertices for each patch (of all 10 parcellations) and subjects. The HC group served as the reference group. Then, each subject's patch (both for patients and HC) was compared against that reference group using nonparametric statistical permutation testing with in‐house software written in MATLAB, version R2022b (The MathWorks), correcting for FWE (Nichols & Holmes, 2002). In brief, we started by computing *z*‐scores for each patch and patient, by rating each of a given patient's patch versus those of the HC group. These *z*‐scores were then converted to *p*‐values using a permutation procedure to correct for the FWER: In brief, we shuffled the data (combing the patient and their controls) under the null hypothesis of no difference between HC and patient and calculated the respective *z*‐scores on each iteration (for each patch separately to account for the physiological differences of thickness across the cortex, Tahedl, 2020). For each patient, we applied an exhaustive permutation procedure, combining all 89 HC and the respective patient. This resulted in a personalized, nonparametric null distribution for each patient and patch. The *p*‐value was then defined as ratio between all *z*‐scores in the null distribution smaller than the observed patient's *z*‐score and the number of iterations. We set the *p*_FWER_‐value to *p*_FWER_ ≤ .05.

### 2.6 Assessing clinical relevance for individual data

One major focus of this study was to evaluate which of the analyzed strategies for assessing GM variations yielded clinically relevant information on an individual patient level. As such, we set up a correlation analyses which analyzed the association between the respective analysis strategy (i.e., each combination of GM metrics, smoothing factor and correction method, as well as MAP) and two clinical variables that cover both structural disease burden (by incorporating LV as DV), as well as functional deficits (by considering EDSS scores as DV). We set up separate correlation analyses for the GM metrics/clinical variables. Notice that to quantify GM changes for a single subject, we used a scalar for each patient as metric of interest which was simply the ratio of all voxels/vertices (smoothed data) or patches (MAP) which were found to be significantly “thin”—suggestive of atrophy—by the above‐specified methods (notice that the reference for that ratio calculation was the cortical mask shown in Figure 2a).

To assess differences between the alternative methods employed, we ran analyses of variance (ANOVA) in each step of the analysis stream as shown in Figure 1, that is, we tested three specific hypotheses with regard to correlation with the dependent clinical variables LV/EDSS: (1) GM changes are not equally correlated to LV and EDSS (testing for differences of correlations with the dependent variables, regardless of the strategy for computing GM changes). (2) There is a difference of the “approach” (testing for differences of [any] standard approach vs. [any] MAP parcellation scheme). (3) Smoothing/parcellation in general makes a difference (testing no smoothing vs. any smoothing for the standard approaches and no parcellation vs. any parcellation for MAP).

Where applicable, we followed up significant ANOVA results in post hoc pairwise comparison using Tukey's Honest Significant Difference (HSD) tests with adjusted *p*‐values, correcting for FWER. These correlation analyses, ANOVAs, and Tukey HSD tests were all run within *RStudio* (using R version 4.2.2, 2022, RStudio Team, 2022), which was also used for figure conception.

### 2.7 Data availability statement

All presented group‐level outputs, statistical maps, variable distributions, post hoc statistics, and additional information on data processing pipelines can be requested from the corresponding author. However, individual patient clinical and neuroimaging data cannot be made available.

## 3 RESULTS

### 3.1 Demographics

Adequate age‐matching of the RRMS and HC group was suggested by a nonsignificant comparison of age means between the two groups using the Welch two‐sample *t*‐test (*t*(103.16) = 1.67, *p* = .10, see Table 1). Similarly, the distribution of gender among the study groups did not change significantly in a Chi‐square test (*χ* ^2^(1, *N* = 554) = 3.27e^−5^, *p* = .995). The mean disease duration in the RRMS group was 8.65 years (SD ± 5.16). RRMS patients had thinner cortices as opposed to HC (*t*(113.31) = −4.60, *p* < .001). LV was on average 5.39 mL (SD ± 8.41 mL) in the RRMS group and the median EDSS score was 1.50 (IQR = 2.00), suggesting a relatively preserved patient group from a clinical perspective.

### 3.2 Qualitative group comparisons suggest high sensitivity of cortical thickness to detect gray matter changes

**FIGURE 3:**
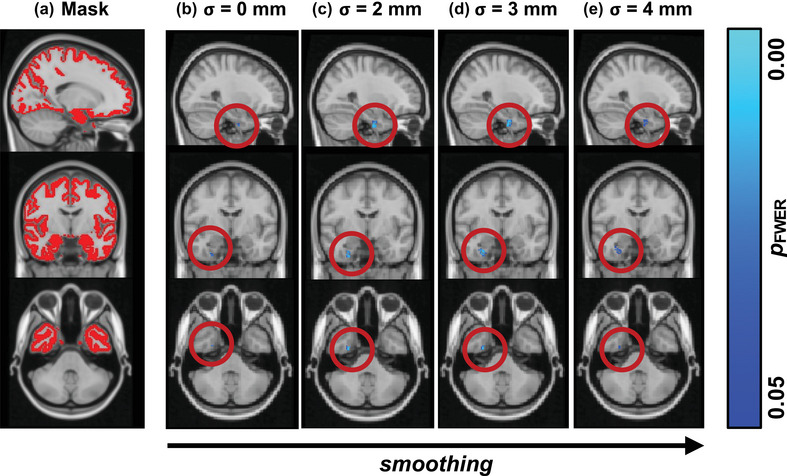
**Group‐level comparison of voxel‐based morphometry (VBM)**. Contrasting VBM between relapsing‐remitting multiple sclerosis (RRMS) and healthy control (HC) for different neuroimaging parameters, we found that false discovery rate (FDR)‐correction (not shown) suppressed any significant results for all levels of smoothing. Therefore, we only show family‐wise error (FWE)‐corrected maps here. Part (a) shows the cortical mask to which we limited our statistical comparisons. All levels of smoothing suggested that a region in the deep right temporal lobe around the parahippocampal gyrus was affected in RRMS as compared to HC. However, although no smoothing (b) and extreme smoothing (e) showed relatively little significant voxels, moderate smoothing (c and d) yielded more clearly defined problematic regions in RRMS. The statistical maps are show on the MNI152 2 mm standard template at voxel location 36‐60‐17. GM, gray matter; TFCE, threshold‐free cluster enhancement.

**FIGURE 4:**
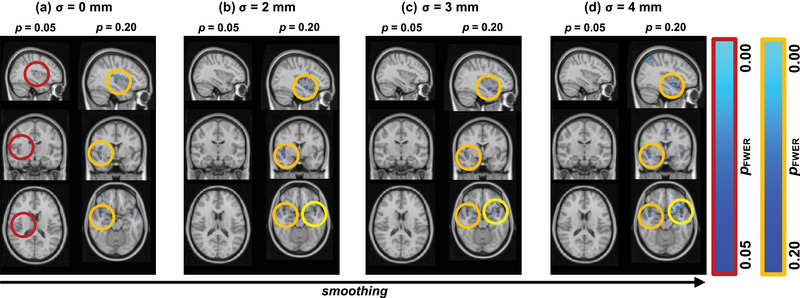
**Group‐level comparison of gray matter (GM) content**. Contrasting CAT12 between relapsing‐remitting multiple sclerosis (RRMS) and healthy control (HC) for different neuroimaging parameters, we found that false discovery rate (FDR)‐correction (not shown) suppressed any significant results for all levels of smoothing. Therefore, we only show family‐wise error (FWE)‐corrected maps here. However, also FWE‐correction suppressed almost any significant results at the alpha‐level *p* ≤ .05 (left columns in a–d). The only exceptions were a few significant voxels for no smoothing (a) at the right insular cortex (red circle). As we chose a more liberal threshold (*p* ≤ .20, right columns in a–d), we found this same region to be affected for all levels of smoothing, supplemented by more regions in the right temporal/insular cortex (orange circles). However, notice that more smoothing (d) also introduced noisy regions (cf. yellow circles in the axial slices of b–c). The statistical maps are show on the MNI152 2 mm standard template at voxel location 27‐56‐44 for *p*_FWER_ ≤ .05 (left columns) and at 25‐63‐28 for *p*_FWER_ ≤ .20 (right columns). CAT12, computational anatomy toolbox, version 12 (used for calculating GM content); TFCE, threshold‐free cluster enhancement.

Comparing the group contrasts qualitatively between the RRMS and HC groups with respect to the metric used for assessing GM atrophy (i.e., CT: Figure 2, VBM: Figure 3, CAT12: Figure 4), we noted that CT (Figure 2) resulted in most data points of significant changes in the RRMS group throughout the cortex, for all levels of smoothing. VBM suggests GM changes in a very sharply defined region around the right insula (Figure 3), whereas CAT12 failed to yield any significantly different data points for the default alpha threshold (except for a few single voxels at the right insular cortex for no smoothing, Figure 4). Only at a more liberal alpha threshold (*p* ≤ .20) did we find “significant” voxels between the study groups, which roughly matched with the location found in the VBM contrast.

### 3.3 Focused gray matter changes of the right temporal/insular cortices are found using different methods

As noted above, the VBM contrast (Figure 4) suggested a very sharply defined affected GM region in the RRMS group around in right insular cortex. Similarly, the liberally thresholded CAT12 contrast yielded a region close to the one described in VBM with a slightly more lateral location, making borders with the temporal lobe. Also, especially the TFCE‐corrected results of the CT comparison yield focused GM changes around the bilateral temporal/insular cortices; however, the affected areas found with CT contrasts covered wide parts of the cortex, including bilateral parietal, frontal, and visual areas.

### 3.4 Smoothing enhances cleanliness but might have an optimum

In terms of smoothing, the qualitative comparisons of the different group contrasts (cf. columns in Figures 2, 3, 4, respectively) suggested mainly two things: (1) Smoothing, in general, seems to slightly enhance statistical power in that it results in a greater number of statistically different data points, regardless of the metric and/or correction method applied. (2) Statistical power and extent of smoothing seem not to be linearly correlated, that is, ever‐more smoothing does not guarantee higher statistical power. This is evident, for example, in the VBM contrast comparing *σ* = 3 mm, Figure 3c, versus*σ* = 4 mm, Figure 3d. When using *σ* = 4 mm, the significant result constituted fewer data points, and those data points yielded higher *p*‐values than for *σ* = 3 mm. This suggests an optimum of smoothing with loss of statistical power for both increases and decreases of smoothing extent from that optimum.

### 3.5 TFCE‐correction outputs relatively more and cleaner results as compared to FDR

Comparing correction methods for multiple comparisons on assessing GM differences between the groups, we noted that TFCE seems superior in terms of statistical power. Two main observations support this claim: (1) FDR‐corrected *p*‐values for VBM and CAT12 data did not yield any significant data points at all (and are therefore not shown in Figures 3 and 4) and (2) when FDR‐correction did show significant differences as with CT data (Figure 2, right columns), the results were much more spread across the cortex and not as clearly defined as TFCE‐corrected results for all levels of smoothing.

### 3.6 MAP yields qualitatively similar results as compared to standard approaches

**FIGURE 5:**
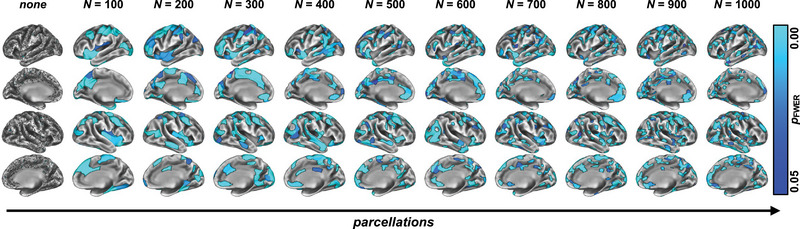
**Group‐level comparison of the mosaic approach (MAP)**. We investigated MAP, a strategy to assess gray matter (GM) variation based on identifying, for single relapsing‐remitting multiple sclerosis (RRMS) patients, which cortical parcels (averaged across all data points constituting that parcel) of a predefined parcellation scheme varied significantly from a healthy control (HC) control group. We ran nonparametric group‐level statistics on these individual statistical maps for 10 different parcellation schemes as well as no parcellation (leftmost column). Although no parcellation produced a rather noisy map, the parceled data suggested GM damage in widespread cortical regions, focused around the bilateral temporal/insular cortices as well as the Somatomotor cortices. Moreover, higher resolution parcellation (i.e., moving rightwards in the figure) produced more clearly defined output maps.

As we inspected the group‐level comparisons of MAP (Figure 5), we made three main observations: (1) As the resolution of the MAP parcellation increased (i.e., the more parcels the cortex was divided into), the results became finer (e.g., compare *N* = 100 in Figure 5 with *N* = 1000). However, no parcellation (i.e., treating each vertex as its own parcel, which is analogous to *N* = 59,234 parcels or “no smoothing”) output a rather random map, which is clinically hard to interpret. Finally, (2) the topography of the affected region stays similar as the parcellation gets finer and is similar to the “standard approach” non‐parceled CT group contrast (Figure 2), with affected regions throughout the cortex including temporal/insular cortices.

### 3.7 MAP yields highest correlations with clinical variables

**FIGURE 6:**
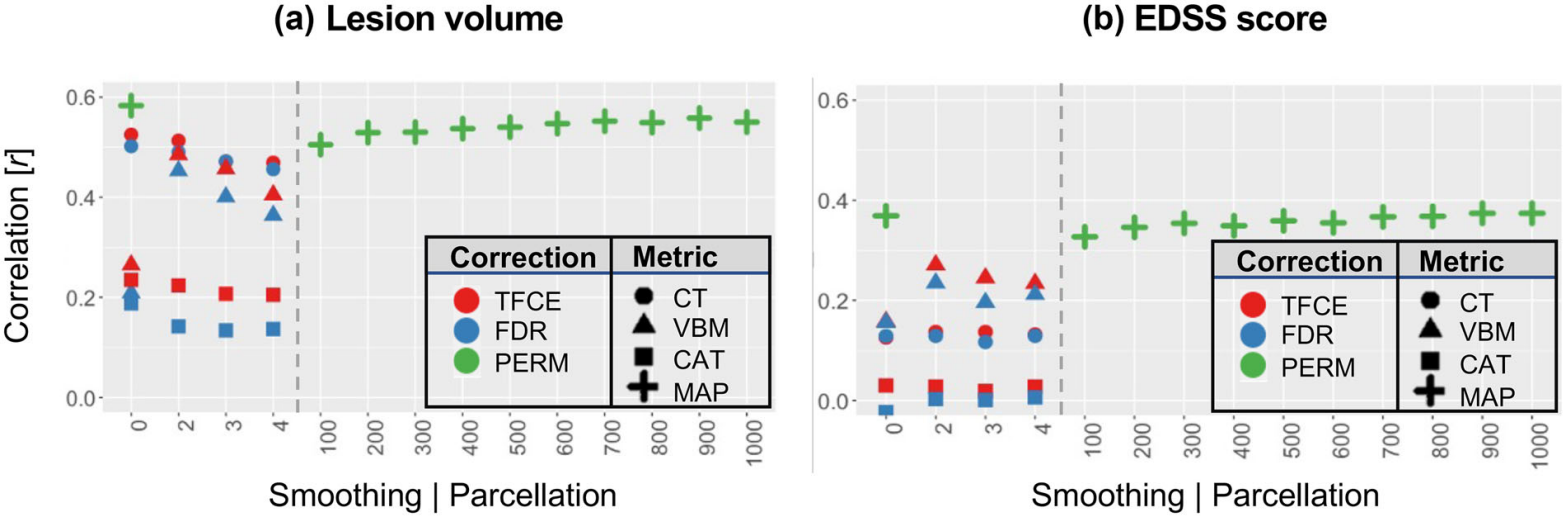
**Comparing methods to assess gray matter (GM) changes for clinical relevance**. We sought to assess which combination of “standard approach” strategies to calculate GM variations (i.e., cortical thickness [CT], voxel‐based morphometry [VBM], CAT12 || smoothing of *σ* = 0/2/3/4 mm || correction for multiple comparisons as threshold‐free cluster enhancement [TFCE] or false discovery rate [FDR]) and/or the mosaic approach (MAP), that is, a method based on parcellating the cortex and calculating which of those parcels deviated from a control population for single patients, yielded highest correlations with two clinically relevant variables, namely, lesion volume (a) expanded disability status scores (EDSS) (b). We found that the MAP yielded higher correlations than any combination of the standard approaches for both lesion volume (LV) (green crosses in a) and EDSS (green crosses in b), whereas the benefits for higher resolution parcellation schemes are neglectable in terms of such quantitative clinical relevance (as opposed to qualitative relevance as demonstrated in this figure, where higher resolution parcellation scheme produces more sharply defined and thus clinically interpretable output maps). Among the standard approaches, family‐wise error (FWE)‐correction (red symbols) produced higher correlations versus FDR‐correction (blue symbols). In terms of smoothing, we found that smoothing in general improved correlations, ever‐more smoothing impaired the results (cf. moving right‐ward on the subpanels). CT (circles) was observed to correlate higher with LV, whereas VBM (triangles) yielded higher correlations with EDSS, and both those metrics exceeded GM content (i.e., CAT12, squares). In general, GM variation was stronger correlated with LV as compared to EDSS scores (compare mean levels of all symbols in a vs. b). CAT12, computational anatomy toolbox, version 12 (used for calculating GM content); HC, healthy controls; RRMS, relapsing‐remitting multiple sclerosis.

One main rationale of this study was to evaluate MAP versus standard approaches for their clinical relevance. We defined “clinical relevance” twofold, namely, (1) LV, to cover structural damage and (2) EDSS, to cover functional deficits. As we compared all combinations of GM metrics/smoothing/correction methods (Table 2), we made three main observations according to our hypotheses (Figure 6, Table 2): (1) In general, GM variation was stronger associated with LV as compared to EDSS score (*F*(3,66) = 41.32, *p* < .001); compare general level of correlations values in Figure 6a vs. b. (2) MAP yielded higher correlations to both LV and EDSS as any of the standard approach combinations (ANOVA: *F*(3,66) = 24.64, *p* < .001; post hoc Tukey HSD: MAP vs. CT: *p*_adj_ = .005, MAP vs. VBM: *p*_adj_ < .001, and MAP vs. CAT12: *p*_adj_ < .001). (3) For the “standard approaches,” there was no statistical difference for smoothing (*F*(1,46) = .388, *p* = .54). Similarly, for MAP, the ANOVA suggested no significant difference for the specific parcellation scheme used (*F*(10,11) = .03, *p* = 1.00).

**TABLE 2:** Correlation values of individual study.

Outcome variable	Gray matter metric	Correction method	Smoothing (sigma)/parcellation number	Correlation (Pearson's *r*)
LV	CT	FWE	0	.525
LV	CT	FWE	2	.513
LV	CT	FWE	3	.471
LV	CT	FWE	4	.469
LV	CT	FDR	0	.502
LV	CT	FDR	2	.49
LV	CT	FDR	3	.472
LV	CT	FDR	4	.456
LV	VBM	FWE	0	.265
LV	VBM	FWE	2	.485
LV	VBM	FWE	3	.457
LV	VBM	FWE	4	.405
LV	VBM	FDR	0	.209
LV	VBM	FDR	2	.453
LV	VBM	FDR	3	.401
LV	VBM	FDR	4	.364
LV	CAT12	FWE	0	.235
LV	CAT12	FWE	2	.224
LV	CAT12	FWE	3	.207
LV	CAT12	FWE	4	.205
LV	CAT12	FDR	0	.188
LV	CAT12	FDR	2	.142
LV	CAT12	FDR	3	.134
LV	CAT12	FDR	4	.137
LV	**MAP**	**Perm**	**0**	**.583**
LV	MAP	Perm	100	.505
LV	MAP	Perm	200	.529
LV	MAP	Perm	300	.53
LV	MAP	Perm	400	.537
LV	MAP	Perm	500	.54
LV	MAP	Perm	600	.547
LV	MAP	Perm	700	.552
LV	MAP	Perm	800	.549
LV	MAP	Perm	900	.558
LV	MAP	Perm	1000	.55
EDSS	MAP	Perm	0	.369
EDSS	MAP	Perm	100	.327
EDSS	MAP	Perm	200	.346
EDSS	MAP	Perm	300	.354
EDSS	MAP	Perm	400	.349
EDSS	MAP	Perm	500	.359
EDSS	MAP	Perm	600	.355
EDSS	MAP	Perm	700	.367
EDSS	MAP	Perm	800	.368
EDSS	**MAP**	**Perm**	**900**	**.374**
EDSS	**MAP**	**Perm**	**1000**	**.374**
EDSS	CT	FWE	0	.126
EDSS	CT	FWE	2	.137
EDSS	CT	FWE	3	.137
EDSS	CT	FWE	4	.132
EDSS	CT	FDR	0	.129
EDSS	CT	FDR	2	.129
EDSS	CT	FDR	3	.117
EDSS	CT	FDR	4	.129
EDSS	VBM	FWE	0	.158
EDSS	VBM	FWE	2	.271
EDSS	VBM	FWE	3	.245
EDSS	VBM	FWE	4	.234
EDSS	VBM	FDR	0	.156
EDSS	VBM	FDR	2	.235
EDSS	VBM	FDR	3	.196
EDSS	VBM	FDR	4	.212
EDSS	CAT12	FWE	0	.03
EDSS	CAT12	FWE	2	.028
EDSS	CAT12	FWE	3	.019
EDSS	CAT12	FWE	4	.028
EDSS	CAT12	FDR	0	−.023
EDSS	CAT12	FDR	2	.003
EDSS	CAT12	FDR	3	.001
EDSS	CAT12	FDR	4	.006

*Note*: Highest correlation values for EDSS and LV are marked in bold.

Abbreviations: CAT12, computational anatomy toolbox, version 12; CT, cortical thickness; EDSS, expanded disability status score; FDR, false discovery rate; FWE, family‐wise error; LV, lesion volume; MAP, mosaic approach; Parc, parcellation; Perm, permutation; VBM, voxel‐based morphometry.

## 4 DISCUSSION

**FIGURE 7:**
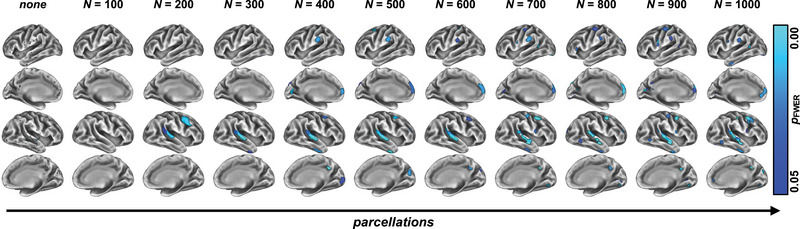
**Visualizing the mosaic approach (MAP) for individual patients**. In addition to the MAP producing more clinically relevant maps of affected gray matter (GM) for single relapsing‐remitting multiple sclerosis (RRMS) patients versus healthy control (HC) (e.g., Figure 6), this method is well‐suited for visualizing individual maps of GM damage. Here, we show the results for a mildly affected 36‐year old female patient (EDSS = 1.5, LV = 0.88 mL). Even for this clinically mildly affected patient, especially the higher resolution maps (i.e., moving rightwards on the figure) suggest established GM damage, focused on the right temporal and premotor cortices. EDSS, expanded disability status score; LV, lesion volume.

In the present study, we sought to compare different neuroimaging strategies for assessing cortical GM variations in RRMS patients as compared to HC. We centered our analysis around a previously suggested method to appreciate individual cortical disease burden in MND and FTD, namely, the MAP, which is based on estimating individual cortical burden with respect to referencing CT data with respect to HC. We evaluated the utility of MAP in MS by comparing its performance against existing approaches to compute individual cortical disease burden (“standard approaches”): Specifically, we investigated three aspects of calculating GM variability, namely, (1) the neuroimaging metric per se (we contrasted three commonly used GM metrics in the neuroimaging community, CT, and GM concentration (via VBM) and GM volume), (2) the smoothing factor (no smoothing and *σ* = 2/3/4 mm), and (3) the correction method for multiple comparisons (FWER vs. FDR corrections). We ran group comparisons between RRMS and HC groups and qualitatively compared the resulting statistical maps. In addition to these “standard approaches,” we also considered MAP, a recently introduced method for assessing GM variation as CT differences on an individual patient's level (Tahedl, 2020; Tahedl et al., 2021), which is based on subdividing the cortex into several subregions, then averaging across all data points constituting that parcel and hence checking for an individual patient whether or not that parcel's observed CT significantly varies from a HC population. In total, we contrasted 10 such parcellation schemes (ranging from *N* = 100 to 1000 parcels in steps of 100, as well as no parcellation). We computed group‐level statistics on the individual patient's parcellations using nonparametric statistical testing and inspected qualitative differences between MAP and the “standard approaches” and effects of more high‐resolution parcellation schemes. Finally, we investigated differences of the analyzed strategies (including all combinations of standard approaches as well as MAP) on correlations to clinically relevant outcome variables on for individual patient's, namely, LV and EDSS. For the (qualitative) group comparisons, we showed that CT produced most sensitive statistical maps, showing widespread GM changes in the RRMS group. VBM and CAT12 only indicated very focused changes around the temporal/insular cortices, which however was only detectable with a more liberal alpha threshold for some combinations. For the individual analysis, it turned out that the MAP method yielded higher correlations than any combination of the standard approaches for both LV and EDSS. However, LV was higher correlated to GM changes as compared to EDSS scores. Although an ANOVA suggested no differences for the specific parcellation scheme applied for MAP, from a qualitative point of view, higher resolution parcellation schemes output sharper defined individual atrophy maps and might therefore be more interesting for clinical interpretation. Another aspect which makes MAP interesting for clinical application is its potential for straightforward visualization of personalized atrophy maps (e.g., Figure 7).

### 4.1 Widespread gray matter atrophy in multiple sclerosis with a focus of right temporal regions

Although this study was primarily of methodological interest, we want to briefly discuss the atrophy patterns identified. In particular, the CT comparisons indicated widespread cortical atrophy in the RRMS patient group as compared to HC. This is in line with previous studies demonstrating both baseline and progressive widespread cortical atrophy in MS (Bergsland et al., 2012; Hidalgo de la Cruz et al., 2021; Narayana et al., 2012; Sailer et al., 2003; Tillema et al., 2016; Tsagkas et al., 2020). Intriguingly, recent work suggests that the topographical patterns of atrophy are nonrandom but that there is an anatomical organization principle to atrophy patterns (Steenwijk et al., 2016). Of note, the locations of WM lesions, more specifically the connected cortical somata, have been found to be strongly correlated (Bussas et al., 2022). Interestingly, we found such widespread differences primarily using CT methods; cortical GM content and volume methods suggested a focus on the temporal cortex. Interestingly, temporal atrophy is related to cognitive decline in MS (Tillema et al., 2016), and—via the association to the limbic system—is affected in the early disease course (Audoin et al., 2010). This is in line with the present study's patient population who yield relatively short disease duration (8.65 years ± 5.16), which suggests that the CT parcellation method is more sensitive to detect subtle differences as opposed to the standard approaches, which might become clinically relevant later on in the disease course.

### 4.2 The potential of MAP as a clinically relevant biomarker in MS and beyond

The main takeaway from this study is that we provide evidence that a clinically relevant biomarker for MS is particularly constructable from parcellated CT data, as realized in the MAP method, which can be (1) easily calculated, (2) applied to, and (3) interpreted for single patients. All of these three characteristics are fundamental for clinical translation of biomarker. Of note, we provide evidence that MAP—that is, referencing single subject's high‐resolution parcellation scheme of the CT data and interpreting distinct patches with respect to a reference group—yields higher correlations with external clinical metrics, namely, LV and EDSS, and might therefore be a promising strategy for quantifying and interpreting GM atrophy in individual patients using MRI. These observations suggest the generalizability of the MAP method—which we have previously shown to be clinically relevant in MND and FTD (McKenna et al., 2022; Tahedl et al., 2021, 2022)—also to MS, where it might help fill the need for a personalized biomarker to appreciate single‐patient cortical involvement (Amiri et al., 2018).

### 4.3 Limits

Although the results of the current study are promising for helping the advancement of MAP a biomarker for estimating cortical disease burden, it comes with certain limits and should therefore be interpreted with caution. First of all, we want to emphasize that we are far from considering all documented strategies and correction methods for assessing GM atrophy and therefore we cannot claim to demonstrate MAP as “the ideal” biomarker. Moreover, our statistical comparisons might suffer from an overall relatively small HC reference group (*N* = 89), which results in discretely distributed *p*‐values in permutation testing, as we did, here, which might in turn hamper statistical power (Nichols & Holmes, 2002). Also note that the original MAP method suggests to further subdivide the HC reference groups into smaller age‐ and gender‐matches subgroups (Tahedl et al., 2021), which we could not provide with the present reference group for its sample size, as outlined above. Note that in the present study, we used an internal reference group to compute MAP, that is, patient and control data were acquired at the same scanner using the same scanning parameters. However, evidence from our previous work suggests that MAP is also valid when using control data from external reference groups acquired using different scanning parameters, such as the HCP (Tahedl, 2020) or the CamCAN repository (Tahedl et al., 2021). Indeed, recent evidence further supports the validity of using external reference data to assess single subject T1w data, which might however require a critical mass of large‐scale control data sets (Bethlehem et al., 2022). Larger and more variable reference groups allow for testing with less discrete distributions of *p*‐values and hence enhance statistical power. However, latter two limits might also be interpreted as a strength of the study's results since the fact that we did find differences suggests strong effect sizes. Moreover, controlling for documented effects on GM estimation related to technical influences, such as TI or the scanner itself (Biberacher et al., 2016; Durand‐Dubief et al., 2012), could not be accounted for in this data set and needs to be further investigated specifically for MAP. Importantly, the reliability of MAP needs to be further evaluated, although some longitudinal reports for this method exist, which at least provide some first evidence of validity of MAP for monitoring clinical progression in ALS and PLS (Tahedl et al., 2021, 2022). Finally, and fundamental for any MS biomarker, is that a metric purely based on cortical disease burden cannot reflect the complexity of the pathology, which—as outlined throughout this manuscript—manifests widespread throughout the CNS, including WM, deep GM, and spinal cord. Nevertheless, we provided evidence that the purely cortical biomarker MAP does transfer clinically relevant information in MS and is therefore a candidate biomarker for monitoring aspects of MS disease state/progression as well as serving as a target for clinical trials. Future studies will need to further refine and investigate MAP to help its translation into daily neuroradiological clinical routine, both for MS and other conditions with cortical involvement—including MND and FTD, for which the method was already adopted—especially in terms of longitudinal reliability and validity, to reach the critical need for a personalized biomarker reflecting single‐patient cortical involvement (Amiri et al., 2018).

## 5 CONCLUSIONS

The present study investigated different methods to assess cortical disease burden for individual MS patients. We found that the mosaic approach or “MAP”—a relatively novel biomarker based on estimating cortical disease burden using CT estimations from high‐resolution parcellation schemes—yields high potential for a clinically relevant biomarker in MS, outperforming existing methods to compute cortical disease burden in single patients. Of note, MAP outputs brain maps illustrating individual cortical disease burden which can be directly interpreted in daily clinical routine.

## AUTHOR CONTRIBUTIONS

**Marlene Tahedl**: Conceptualization; formal analysis; funding acquisition; investigation; methodology; resources; validation; visualization; writing—original draft; writing—review and editing. **Tun Wiltgen**: Investigation; writing—review and editing. **Cui Ci Voon**: Writing—review and editing. **Achim Berthele**: Resources; supervision; writing—review and editing. **Jan S. Kirschke**: Data curation; funding acquisition; project administration; resources; supervision; writing—review and editing. **Bernhard Hemmer**: Data curation; funding acquisition; project administration; resources; supervision; writing—review and editing. **Mark Mühlau**: Conceptualization; data curation; methodology; resources; supervision; writing—original draft; writing—review and editing. **Claus Zimmer**: Data curation; funding acquisition; project administration; resources; supervision; writing—review and editing. **Benedikt Wiestler**: Conceptualization; formal analysis; funding acquisition; methodology; project administration; supervision; writing—original draft; writing—review and editing.

## CONFLICT OF INTEREST STATEMENT

AB has received consulting and/or speaker fees from Alexion, Biogen, Celgene, Horizon, Novartis, Roche and Sandoz/Hexal, and his institution has received compensation for clinical trials from Alexion, Biogen, Merck, Novartis, Roche, and Sanofi Genzyme, all outside the current work. JSK has received speaker fees from Novartis. BH has served on scientific advisory boards for Novartis; he has served as DMSC member for AllergyCare, Sandoz, Polpharma, Biocon, and TG therapeutics; his institution received research grants from Roche for multiple sclerosis research. He has received honoraria for counseling (Gerson Lehrman Group). He holds part of two patents; one for the detection of antibodies against KIR4.1 in a subpopulation of patients with multiple sclerosis and one for genetic determinants of neutralizing antibodies to interferon. All conflicts are not relevant to the topic of the study.

## DISCLOSURES

AB has received consulting and/or speaker fees outside of the current work. The details are at the end of the manuscript. The other authors declare no competing interests.

### PEER REVIEW

The peer review history for this article is available at https://publons.com/publon/10.1002/brb3.3327.

## Data Availability

NA.
